# Editorial: The oral microbiome in an ecological perspective

**DOI:** 10.3389/fcimb.2015.00039

**Published:** 2015-04-29

**Authors:** Egija Zaura, Alex Mira

**Affiliations:** ^1^Department of Preventive Dentistry, Academic Centre for Dentistry Amsterdam (ACTA), University of Amsterdam and Free University AmsterdamAmsterdam, Netherlands; ^2^Department of Genomics and Health, FISABIO Foundation, Center for Advanced Research in Public HealthValencia, Spain

**Keywords:** metatranscriptomics, horizontal gene transfer, fungal–bacterial interactions, quorum sensing, immune system, dental plaque, biofilm, oral ecology

Pure cultures have been the basis for microbiology research over a century. However, although working with clonal lineages in the laboratory has allowed fundamental advances in microbial physiology and genetics, microorganisms are never alone. Even extreme environments like hypersaline waters or acidic ponds are not formed by single species. The human body is no exception, and the oral cavity contains hundreds of bacterial species, that together with fungal and viral inhabitants form highly complex communities where they interact with each other and with the host. These interactions include physical coaggregation, chemical signaling, transfer of genetic information, stimulation of the immune system, metabolic complementation, growth synergism and antagonism or pH buffering among others, and they are so intricate that the final contributing output of the whole community is much larger than the addition of the individual species forming it. This is why the use of holistic, metagenomic approaches to study oral microbial ecology becomes fundamental to understand the ecosystem in health and disease.

The advent of high throughput sequencing techniques has allowed gathering a wealth of data on the bacterial content of the oral cavity. However, most of this work has initially been focused on descriptive studies in which the general taxonomy composition of microbial communities was depicted. We must now enter a second phase in which more functional approaches are performed, including *bona fide* metagenomic and metatranscriptomic approaches in which the total gene repertoire and actively expressed genes in the community are identified under different circumstances, without the biases imposed by PCR or cloning procedures. Experimental approaches are also required to validate the correlations that may have been suggested by taxonomic studies and to describe the molecular basis for inter-species interactions. Finally, we cannot forget the physical environment where oral microbes thrive and where the immune system probably plays a crucial role in selecting for a given community. We believe that understanding the basis for these ecological interactions will provide formidable insights to diagnose oral diseases and to prevent its development, and hope that this special issue may contribute to that purpose.

In this topic two functional studies have been included, in which RNAseq strategy has allowed researchers to describe the mRNA populations of dental plaque in twins, in an attempt to normalize for genetic host factors (Peterson et al., 2014); and to focus on the gene expression patterns of different *Veillonella* species within caries lesions (Do et al., 2015). One of the consequences of the close physical interaction in oral biofilms is the possibility for horizontal genetic transfer (reviewed by Roberts and Kreth, 2014), with important consequences in relation to antibiotic resistance. As Bachtiar and colleagues show, inter-species interactions are not limited to closely related organisms but can actually cross kingdom borders, and the authors describe a surprising case of quorum sensing signals produced by a gram-negative bacterium that inhibits biofilm formation in *Candida* (Bachtiar et al., 2014). In fact, the ecology of fungal–bacterial interactions may be instrumental for development of oral biofilms. Microbiome studies have been severely biased toward the prokaryotic component, assuming that fungal species only play a role as opportunistic pathogens. This view is changing fast, and the potential relevance of bacterial–fungal interactions to oral health is now being evaluated as shown by the review by Diaz et al. (2014).

The new technical possibilities in unraveling the complexity of the oral ecosystem are brought forward by McLean, where advancements toward a systems level understanding of the human oral microbiome are presented (McLean, 2014). An applied aspect of ecological interactions is shown by studying the effect of an oxygenating agent on oral microorganisms *in vivo*, where twice daily exposure to this agent in the form of a mouthwash prevented plaque growth and induced shift in microbial composition (Fernandez y Mostajo et al., 2014).

The role of indigenous microbiome in maintaining oral health has been addressed by Kumar and Mason, where the interaction of the microbiome and the host receives the attention (Kumar and Mason, 2015). Continuing along the same lines on the interaction of microbes with the immune system, Zaura and collaborators propose an interesting hypothesis about how a normal oral microbiome is acquired (Zaura et al., 2014). Their view is that fetal tolerance toward the mother's microbiota during pregnancy is a major factor selecting for the acquisition of the oral microbiome.

There is no doubt that the next generation sequencing (NGS) technologies have revolutionized the field of microbiology. However, the price to pay for the high throughput output is low taxonomical accuracy and sequencing bias. Schulze-Schweifing and colleagues compare the different approaches for microbiome characterization: culture, traditional cloning and sequencing as well as high throughput sequencing (Schulze-Schweifing et al., 2014), while Lazarevic and colleagues demonstrate the difficulties in work with low DNA yield samples (Lazarevic et al., 2014).

The overall purpose of the studies on ecological interactions of oral microbial communities is to be able to apply that knowledge to understand and prevent oral diseases. In this direction, Rosier and colleagues present a comprehensive and helpful review on the historical hypotheses that have attempted to explain the development of oral diseases (Rosier et al., 2014). With this topic we have summarized the current insights and identified the goals for future research in oral microbial ecology. We believe the field will benefit enormously from these ecological approaches, which certainly show that oral microbial communities cannot be understood by the isolated study of their individual microorganisms and that they are much more complex than the addition of its microbial and host components.

## Conflict of interest statement

The authors declare that the research was conducted in the absence of any commercial or financial relationships that could be construed as a potential conflict of interest.
